# Recognition of Social Rule Violation in “Deficit Syndrome” Schizophrenia: A Study Using Economic Games

**DOI:** 10.3389/fpsyt.2020.00835

**Published:** 2020-08-21

**Authors:** Christian Claassen, Robyn Langdon, Martin Brüne

**Affiliations:** ^1^ Klinikum Osnabrück, Klinik für Neurologie und neurologische Frührehabilitation, Osnabrück, Germany; ^2^ Department of Cognitive Science and ARC Centre of Excellence in Cognition and its Disorders, Macquarie University, Sydney, NSW, Australia; ^3^ Department of Psychiatry, Psychotherapy and Preventive Medicine, Division of Social Neuropsychiatry and Evolutionary Medicine, LWL University Hospital, Ruhr University Bochum, Bochum, Germany

**Keywords:** social rules, deficit syndrome, schizophrenia, neuroeconomic games, theory of mind

## Abstract

Aberrant social behavior is a frequent clinical feature of schizophrenia and seems related to the duration and chronicity of the disorder. However, there is a paucity of research into the relationship between social behavior and social cognition in patients with severe chronic courses of schizophrenia. Accordingly, the present study sought to examine the appreciation of social rules and norms such as fairness and cooperation in schizophrenia patients who fulfilled the criteria for “deficit syndrome”. To this end, we utilized a so-called Ultimatum Game, and a Dictator Game, in which participants had the option to punish others’ unfair behavior. In addition, “theory of mind”, the ability to appreciate others’ mental states, was also examined using the Mental State Attribution Task (MSAT). Symptom severity was determined using the Positive and Negative Syndrome Scale. While patients with deficit schizophrenia responded to varying levels of fairness in similar ways to controls, the patients accepted fewer fair offers and engaged less in third-party punishment. Impaired theory of mind in patients reduced the latter, but not the former, group difference to non-significance. No significant correlations emerged between symptom severity and task performance. Together, these findings suggest that the understanding of others’ minds partly contributes to the appreciation of social rules and norms in patients with severe chronic courses of schizophrenia.

## Introduction

The term “schizophrenia” refers to a group of severe mental disorders that is characterized by delusions, hallucinations, disorganized speech and behavior, anhedonia, apathy, and social dysfunction (1). Social dysfunction is often associated with compromised social cognition that seems to affect social functioning independently from non-social cognition (2). One important component of social cognition is theory of mind (ToM), the ability to reflect upon thoughts, intentions, desires and emotions of oneself and others (3), sometimes interchangeably used with the term “mentalising” [e.g., (4)]. ToM is known to be impaired in schizophrenia, and it might be linked to the pathophysiology of psychotic symptoms and act as a predictor for social functioning (5, 6).

Another important factor related to poorer social functioning in schizophrenia concerns patients’ ignorance of more complex social norms and rules, as noted in Hecker’s (7) early description of “hebephrenia”. More precisely, Hecker described hebephrenia as often taking a chronic deteriorating course with transgressions of social etiquette being typical for this subtype of psychosis.

In spite of these early hints toward impaired recognition of “moral” values, only a small body of research, primarily from the 1960’s to 1980’s, has addressed this important feature of psychosis empirically. For example, in one study using a semi-structured interview, The Tsedek Test of Moral Judgement, it was shown that, while healthy controls took a rather humanitarian approach to moral issues, patients with schizophrenia considered authoritarian and self-protective values to be more important (8). Furthermore, when confronted with hypothetical situations requiring moral decisions, schizophrenia patients tended to display less stable ideas on morality than controls (9). Other research using the Kohlberg Moral Judgement Interview (MJI) reported that adolescents with schizophrenia employed “less mature (…) moral reasoning” when justifying moral judgments, suggesting difficulties in explaining the process of how to arrive at a certain judgement. Instead, they considered concepts as “power, status, and possessions” as more important than “equality, reciprocity, and trust” (10). In a more recent search for an explanation for these early findings on moral reasoning using the *MJI*, it has been argued that these studies might have underestimated confounding factors like social cognitive deficits and psychopathic personality traits and thus led to a skewed view on moral cognition in schizophrenia (11). This interpretation is supported by evidence suggesting that social cognitive deficits seemed to partially account for patients’ poor performance in the *MJI* (12). In other recent work on moral judgment, rather than moral reasoning, patients with schizophrenia have also been found to employ more utilitarian (or outcome-focused) decision-making than healthy controls when asked to judge whether it is morally appropriate for an agent in a moral dilemma scenario to harm one to serve the greater good (13).

A related, though in part diverging approach to study social norm recognition and social decision-making in schizophrenia, has utilized neuroeconomic games to model interactive situations in which players are required to act on the violation of a commonly accepted fairness rule (e.g., “tit-for-tat”). Abundant research has demonstrated that psychologically healthy individuals often chose seemingly “irrational” altruistic responses (even if the declared aim of the game entails maximization of one’s own benefit). Individuals with schizophrenia, in contrast, show somewhat aberrant responses, though fairly inconsistent. Specifically, in a so-called Ultimatum Game (UG) (14, 15), schizophrenia patients accepted significantly more unfair offers and rejected significantly more fair offers than healthy controls, even though overall, patients’ acceptance of unfair offers declined with growing unfairness, akin to what has been found in unaffected control samples (16–19). Along similar lines, work on altruistic punishment of another’s unfair behavior (20, 21) revealed that healthy individuals tend to invest own monetary resources to re-establish fairness, which has turned out to be similar in a group of patients with schizophrenia (16). Notably, economic decision-making was found to be largely unrelated to impaired ToM in schizophrenia.

Taken together, while the appreciation of fairness rules in schizophrenia appears to remain intact to some degree, it is unclear to what extent clinical characteristics, including chronicity of the disorder, might compromise this social understanding. This question is worthy of study, because schizophrenia patients with a so-called “deficit syndrome” show distinct socio-cognitive deficits including limited emotional responsivity and problems in dealing with complex social situations (22, 23). Deficit schizophrenia is also associated with deviant discrimination of facial affect (24), poor ToM compared to non-deficit patients (25), as well as diminished empathy and self-confidence (26).

Accordingly, the present study aimed to examine whether patients with deficit syndrome would display difficulties in appreciating fairness rules and whether performance in economic games was dependent on ToM abilities.

## Methods

### Participants

Thirty patients (10 female and 20 male) with a diagnosis of schizophrenia according to DSM-IV-R criteria (i.e., the presence of at least two of the following: delusions, hallucinations, grossly disorganized or catatonic behavior, negative symptoms, i.e., affective flattening, alogia, or avolition) participated in the study. In addition, they all met the concept of deficit schizophrenia, a subtype of schizophrenia that features negative symptoms over a prolonged duration (usually 12 months or more) as a stable trait (27). Following the work of Bryson and colleagues, a duration of psychosis of eight or more years was also required (22, 28). In our sample, patients’ mean duration of psychosis was 19.3 years. All patients had been followed-up for many years in the out-patient clinic of the LWL University Hospital Bochum and were on stable doses of second-generation antipsychotics. The severity of psychopathology was rated by an experienced clinician (blind to participants’ performance in the other tasks) using the Positive and Negative Syndrome Scale [PANSS; (29)]. Accordingly, patients were moderately ill with a PANSS positive syndrome score of 18.7 (SD 7.3), negative syndrome score of 17.1 (SD 8.0), a general psychopathology score of 32.0 (SD 13.0), resulting in a total PANSS score of 67.8 (SD 22.0).

For comparison, 30 healthy subjects (20 females) were recruited from the general public and the local university. Patient and control groups were similar in age and education. Drug abuse (except for tobacco), severe neuropsychiatric (other than schizophrenia) or somatic illnesses, mental retardation or insufficient knowledge of German language were exclusion criteria. All participants gave informed consent to participate in the study. The study was approved by the Ethics Committee of the Medical Faculty of Ruhr-University Bochum, Germany.

The patients’ mean age was 42.8 (SD 10.28) with a verbal IQ of 101 (SD 13.42) (as measured using the Mehrfachwahl-Wortschatztest (MWT-B), a common German screening instrument for verbal intelligence, which is similar to the Spot-the-Word test (30). The control group had a mean age of 42.8 (SD 13.74) with a slightly higher mean verbal IQ of 108 (SD 15.37). This difference in IQ was marginally significant (t = 1.98, df = 58, p = .053). Demographic and clinical characteristics are summarized in **Table 1**.

**Table 1:** Demographic data and psychopathology ratings of patients with schizophrenia and controls.

	Schizophrenia	Controls
N	30	30
M:F ratio Age Duration of illness	20:10 42.8 (10.3) 19.3 (9.2)	10:20 42.8 (13.8) —
Verbal IQ	101 (13.4)	108 (15.4)
PANSS positive	18.7 (7.3)	—
PANSS negative	17.1 (8.0)	—
PANSS global	32.0 (13.0)	—
PANSS sum score	67.8 (22.0)	—

M, male; F, female; PANSS, Positive and Negative Syndrome Scale.

### Tasks

#### Economic Games

The economic games used here were adapted versions of the ones used by Wischniewski and Brüne (16). Prior to testing, all participants were provided with written and oral instructions and performed a practice trial. Participants received 10 Euros for participation, and another 0 to 5 Euros depending on their actual performance in the tests. All participants were informed about the possibility of gaining additional money but not about the exact mathematical procedure according to which the money was distributed. Thus, they did not know whether altruistic or selfish behavior was rewarded. In fact, participants received an additional 10 percent of the money invested in punishment in the Dictator Game; hence, they were “rewarded” for altruistic punishment.

##### Ultimatum Game

The UG is a neuroeconomic task requiring an understanding of fairness. The setup of the UG is such that two players are asked to decide how to split a fixed amount of money or money units (MU). One player assumes the role of a proposer, the other acts as a (passive) recipient. In our version of the UG, participants played the role of the recipient. The proposer (a virtual character) suggests to the recipient how to split 10 MU (depicted by a € symbol). There were three trials per split condition with shares of 5:5, 7:3, 8:2, and 9:1, respectively. We decided to shorten the original version developed by Wischniewski and Brüne (16), as a pre-test with the original 44-trial version (i.e., 11 trials per split condition) revealed that the chronic patients found the task taxing, most likely due to a substantially reduced attention span. The trials were shown in a random order. Thus, there was one fair condition in which the proposer offered 50 percent of the total MU and three other conditions varying in their degree of unfairness. Participants first viewed a picture of a face of a virtual person who would make the offer. Then, the proposer’s offer was shown on a computer screen. Subsequently, the participants were asked to decide whether they would accept or decline the given offer by clicking a mouse-button as quickly as possible (rejecting the offer resulted in a complete loss for both, acceptance led to an outcome according to the proposal). In other words, rejecting an unfair offer implied a mild form of punishment of the proposer, however at the cost for the recipient of losing some MU. Thus, strictly speaking, the most “rational” decision (from an economic point of view) would be to accept any offer. To ensure that participants acted as similar as possible as in “real-life” encounters, they were told that the facial images of the proposer were placeholders for real persons who had acted in exactly the same way as in previous games. We calculated mean acceptance rates per condition in percent.

##### Dictator Game With Punishment Option

The Dictator Game with the option to punish observed unfairness (DG-P) introduced a third character. That is, two virtual players (proposer and recipient) played a Dictator Game. This is similar to the UG, except that the recipient has no option to reject unfair offers. Instead, a third player (the participant) was given 10 MU per round and had the opportunity to re-install equity at his or her cost by investing some of his or her MU at their choice (in 0.5 MU steps). For every 0.5 MU invested to punish the proposer, the proposer’s amount was reduced by 1 MU while the recipient’s amount increased by 1 MU. For example, if the proposer made the offer to keep 8 MU for himself and to give 2 MU to the recipient, the participant, in the role of a third-party player, might invest 1.5 MU, which would deduce 3 MU from the proposers’ sum, and add 3 MU to the recipient’ amount, thus inducing equity (or fairness) in this example.

**Figure 1:**
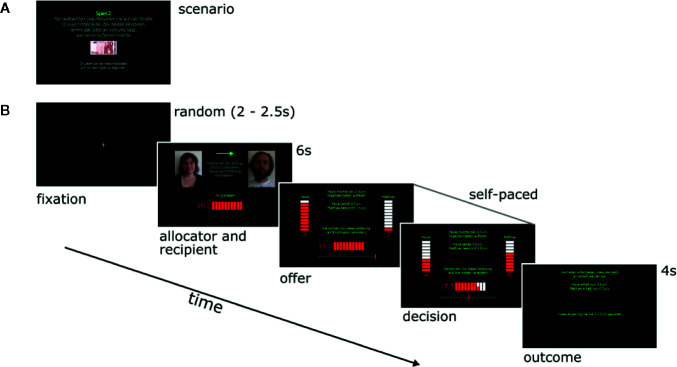
**(A)** Introductory screen to the Dictator Game with Punishment Option (DG-P); **(B)** timeline of screens within an exemplary trial of the DG-P. (1) a fixation cross indicating the beginning of a trial (duration time, 2–2.5 s); (2) the proposer and recipient are introduced with their facial images and names (duration time, 6 s); (3) the third and fourth screen show the dynamic process of decision-making when moving the slide bar in the lower part of the image; (4) feedback concerning the final outcome (duration time, 4 s).

Similar to the UG, there were a total of 12 trials with 3 trials per split condition (5:5, 7:3, 8:2, and 9:1). The trials were presented in random order. Participants first viewed facial images of the two players (proposer and recipient), as in the UG, and were then shown the amount that the proposer offered to the recipient. In the next step, each participant was asked whether, and if so, to what amount he or she would like to change the distribution by investing some of his or her 10 MU. The distribution of MU was visualized using a slide bar and stacks of MU, such that no mathematical calculation was required. The participants moved a computer mouse cursor to the left or right, changing the invested MU and observing in real-time the impact of his or her decision on the other two players’ MU. Finally, the participant confirmed the invested amount with a mouse click and was shown a summary displaying the respective payoffs of Player A and B and the participant’s punishment investment (the setup of the DG-P is illustrated in **Figure 1**).

#### Theory of Mind

**Figure 2:**
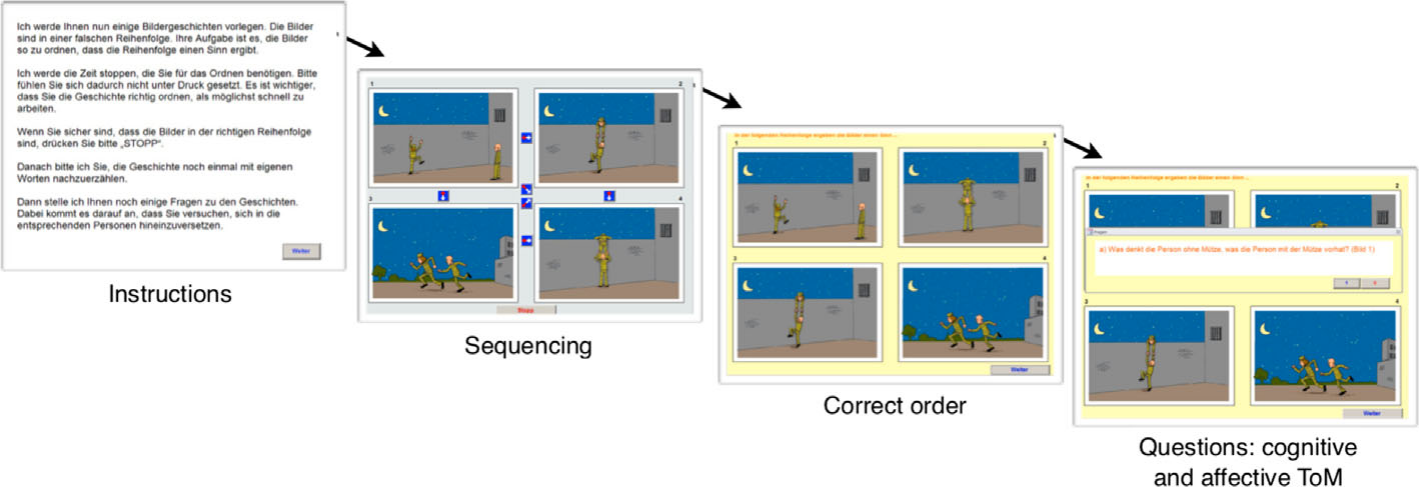
Illustration of the ToM task. Following written instructions, cartoon images are presented in jumbled order. Participants are asked to re-order the images using the cursor. Correct sequencing performance requires accurate inferences of the story characters’ mental states. In case the cartoon story is incorrectly sequenced, the right order is presented before questions about cognitive and affective aspects are asked.

The ability to infer another’s thoughts or intentions was tested using a computerized version of the Mental State Attribution Task (MSAT), comprising a picture sequencing task and a questionnaire [(6) see **Figure 2**]. It consists of six cartoon stories, two of which show two persons cooperating with each other, two other cartoons showing one character deceiving another, and two cartoon stories depicting two people cooperating to deceive a third person. The participants were given 6 points for sequencing a story correctly (thus, maximum score for sequencing was 36 points) and a maximum of 23 points for answering questions about the characters’ mental states in terms of thoughts, beliefs, and intentions (thus, total score maximum was 59 points). The MSAT has been used in behavioral and neuroimaging studies from our own group [e.g., (31–34)]. It has also been translated in several languages, including English, Portuguese, Italian, and Chinese, and utilized in schizophrenia research, including the effects of oxytocin on social cognition [e.g., (35)].

#### Statistical Analysis

Statistical analysis was performed using the Statistical Package for the Social Sciences (SPSS), Version 26 for Windows. In line with previous research on analyses of mean count data (36), we first calculated the percentage of the mean acceptance rate (in the UG) and the invested MU for each offer (in the DG-P). We then used multivariate analyses of variance (MANOVAs) to compare differences in performance on the UG and DG-P between the groups with the four conditions (5:5, 7:3, 8:2, and 9:1) as the dependent variables (DVs). We also report results of ANOVAs comparing groups for each separate DV. The reason for choosing this statistical approach was that the data deviated from normality in some conditions. MANOVAs are considered fairly robust against violations of normality. To test whether performance in economic games was dependent on ToM abilities, any significant group differences in game performance were then followed-up using MANCOVAs, and ANCOVAs, as appropriate, with total ToM performance as a co-variate. We also used one-way ANOVAs for group comparisons of ToM scores, as well as ANCOVAs (to control for IQ). Because most clinical measures used ordinal scales, spearman-rho correlation analyses examined relations between task scores and clinical measures. Given the number of correlational analyses, we adjusted alpha to 0.01.

## Results

### Between-Group Differences

#### Theory of Mind

Schizophrenia patients had significantly lower ToM scores than controls. That is, they performed more poorly in the sequencing task than controls (26.9 ± 8.2 versus 32.3 ± 4.8 points; F = 9.622; df = 1,58; p **= .**003; η_p_^2^ **= .**142) and in the questionnaire part (18.4 ± 5.2 versus 22.3 ± 1.3 points; F = 15.927; df = 1,58; p <.001; η_p_^2^ **= .**215), resulting in a significant difference in the total ToM score (45.3 ± 12.1 versus 54.6 ± 5.6 points; F = 14.505; df= 1,58; p <.001; η_p_^2^ **= .**200). Due to marginally significant differences in IQ (as reported above), we controlled group comparisons for IQ, showing that ToM differences remained highly significant (e.g., for the total ToM score, F = 10.217; df = 1,57; p **= .**002; η_p_^2^ **= .**152).

#### Neuroeconomic Games

##### Ultimatum Game

**Figure 3:**
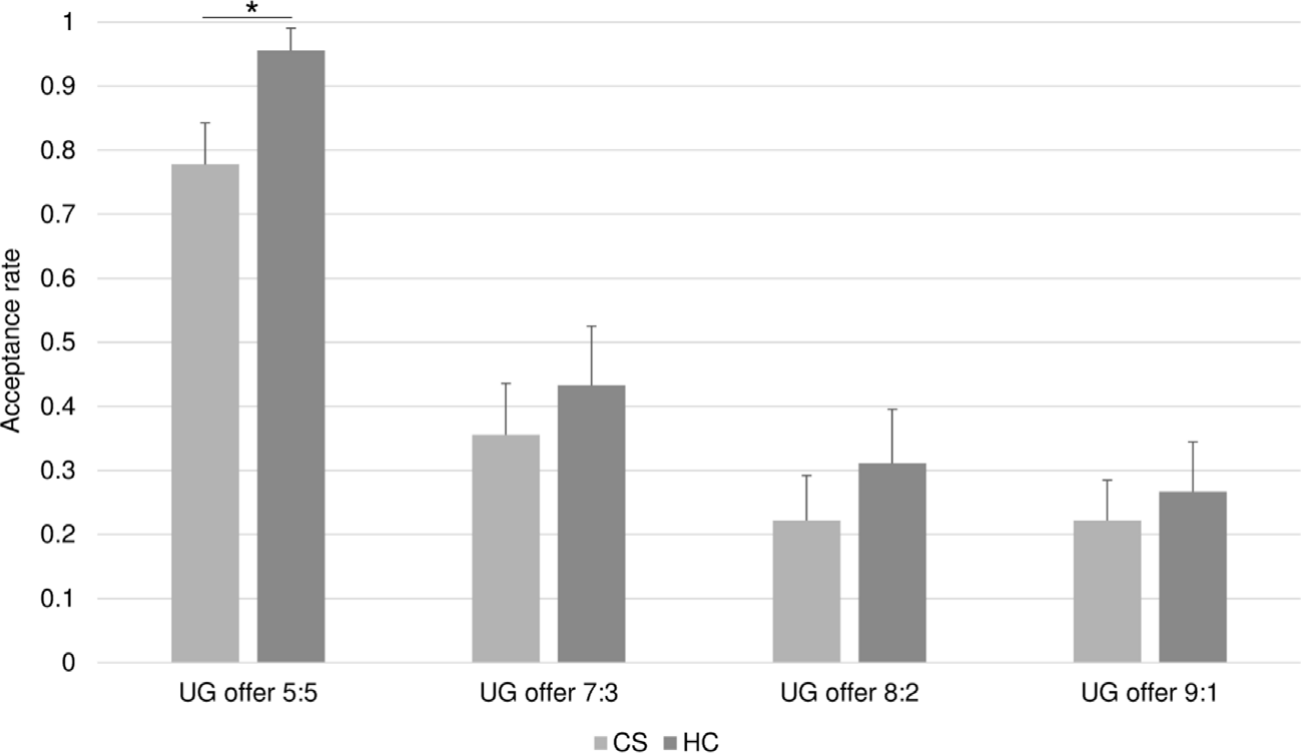
Acceptance rates in the UG. Bars represent the proportion of accepted offers (y-axis) in the four split conditions (x-axis) for the healthy controls (HC) and the chronic schizophrenia patients (CS). Overall, acceptance rates decline with the degree of unfairness of the offer in both groups. The CS group accepts significantly less offers in the fair 5:5 split condition. *p < 0.05.

Findings, as depicted in **Figure 3**, illustrate that both groups showed decreasing acceptance rates as offers became more unfair. A MANOVA with the four split conditions as DVs and diagnosis as the independent variable (IV) revealed a non-significant overall effect of diagnosis (F = 1.454; df = 4,55; p = .229; η_p_^2^ = .095). The pattern of ANOVA results was slightly different in showing that the group difference for the fair split (i.e., 5:5) condition reached statistical significance (F = 5.873; df = 1.58; p = .019; η_p_^2^ = .092), while ANOVA results for all other DVs were non-significant (all *p*’s <.42; all η_p_^2^ <.011). An ANCOVA comparing groups for the fair split (i.e., 5:5) condition showed that the patients still continued to reject significantly fewer fair offers, after adjusting for ToM (F = 7.323; df = 1.57; p = .009; η_p_^2^ = .114). Since the overall MANOVA was non-significant, we tested the robustness of this latter result using binary logistic regression analysis to predict group membership with ToM and acceptance of fair offers as predictor variables. Results were consistent with the ANCOVA results; the full model was highly significant (*χ* ^2^ = 21.34, *p* < 0.0005) and both ToM and acceptance of fair offers were significant independent predictors of group membership (change in Log-Likelihood if ToM removed from the model = 15.46, p < 0.0005; change in Log-Likelihood if acceptance of fair offers removed from the model = 6.16, p = 0.013. In other words, results suggested that poorer ToM in patients did not fully account for these individuals’ reduced acceptance of fair offers in the UG and suggested instead the involvement of other distinct factor(s).

##### Dictator Game With Punishment Option

**Figure 4:**
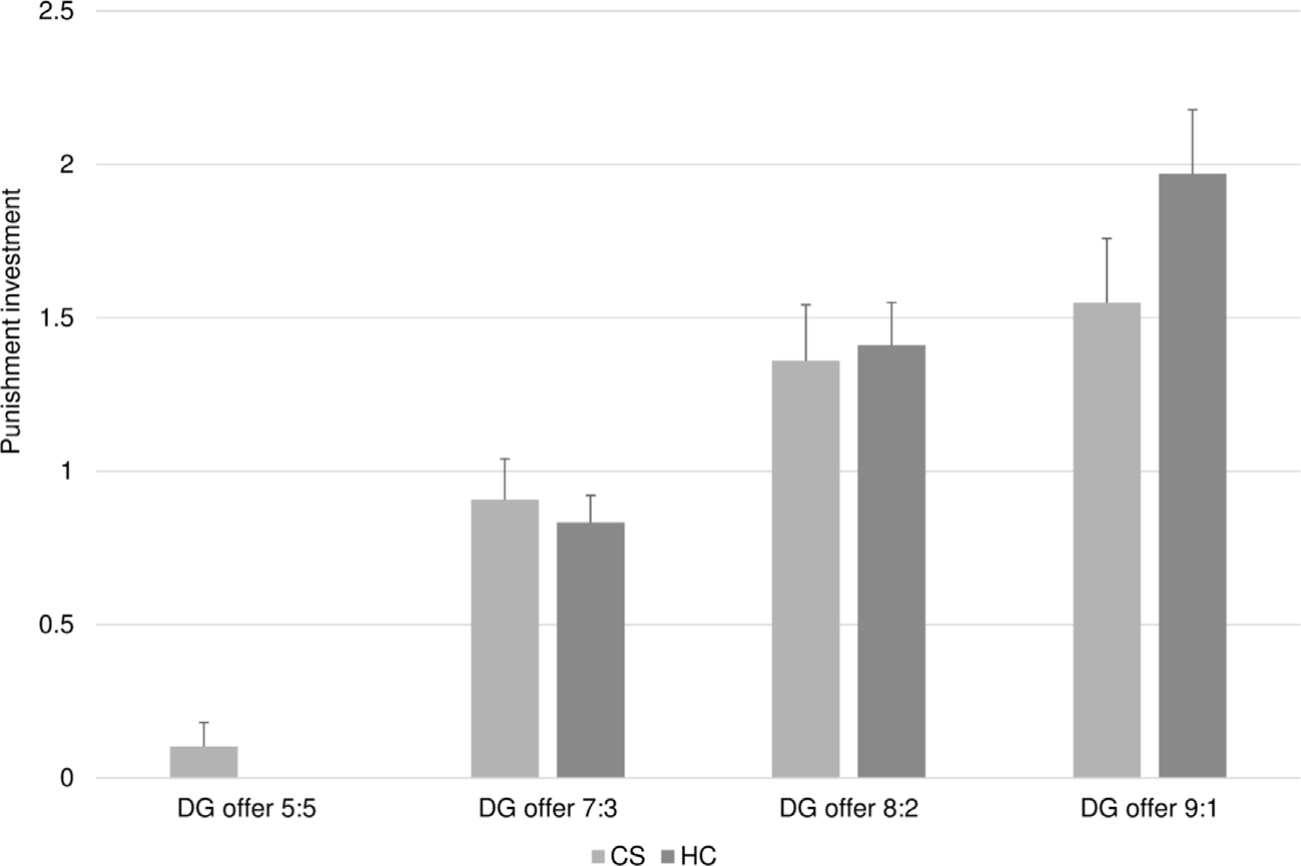
Average punishment investment in the DG. Bars represent the average amount of money units invested (y-axis) in the four split conditions (x-axis) for the healthy controls (HC) and the chronic schizophrenia patients (CS). Punishment investment increased with growing unfairness of the offers in both groups. Overall, the CZ group invested less in third-party punishment.

As regards the DG-P, **Figure 4** shows that both groups invested more in punishment as behavior became more unfair. A MANOVA with punishment investments for each condition as the DVs and diagnosis as the IV showed a significant overall effect of diagnosis (F = 2.560; df = 4,55; p = .049; η_p_^2^ = .159). No ANOVA results for the separate DVs were significant (all *p*’s >.15; all η_p_^2^ <.034). When introducing ToM as a covariate, the MANCOVA revealed a significant effect for ToM (F = 3.091; df = 4,53; p = .023; η_p_^2^ = .189), whereas the effect of diagnosis no longer remained significant, albeit continuing to show a trend (F = 2.322; df = 4,53; p = .069; η_p_^2^ = .149). In other words, after adjusting for the significant effect of poorer ToM in patients, the group difference for investment in third-party punishment of unfairness was no longer statistically significant.

### Correlations Within the Patient Group

Spearman-rho correlations examined associations between cognitive task scores, performances in the neuroeconomic games, illness duration, and symptom severity. While no correlations between duration of illness and other scores were statistically significant at a 0.01 alpha level, there was a non-significant tendency toward an inverse correlation with ToM (r_s_ = −.381, p = .042), consistent with the literature. As regards ToM, there was an additional correlation between ToM total score and verbal IQ (r_s_ = .421, p = .001), but not with any one of the economic game results. With regard to psychopathology scores, there was only one significant inverse correlation between PANSS negative score and verbal IQ (r_s_ = −.548, p = .007), but no significant associations with neuroeconomic decision-making.

## Discussion

Schizophrenia is a heterogeneous group of disorders with marked impairment of social functioning (37). While antipsychotic drugs have the potential to reduce positive symptoms, a subgroup of patients with so-called “deficit syndrome” is characterized by the absence of remission, persistent negative symptoms and relative unresponsiveness to antipsychotic medication (1, 23, 38).

One consistent finding in the literature is that patients with schizophrenia have difficulties in social cognitive task performance, including affective face and voice perception, ToM or mentalising, and emotion recognition, which independently contribute to poor social functioning (39). Deficits in social cognition and aberrant neurocognition are known to affect social functioning (40). To our knowledge, there is a paucity of research addressing social cognition in the most severely and chronically ill patients with schizophrenia (24, 25).

Accordingly, we sought to study patients’ ability to comprehend basic rules of social exchange and fairness and to explore whether this kind of economic decision-making was related to social cognitive abilities such as ToM or duration of illness. Our hypotheses were partly confirmed. Patients with deficit syndromes had some basic understanding of fairness rules and equity, as shown by similar incremental rejection of unfair offers in a UG, and third-party punishment of observed unfairness. However, similar to a multitude of studies in patients with schizophrenia, individuals with “deficit syndrome” performed more poorly on a cognitive ToM task compared to controls, though comparable to our own previous work using the same ToM task in different samples with schizophrenia that did not fulfil the criteria for “deficit syndrome” (6, 31, 33, 41).

In contrast to a previous study in schizophrenia with shorter disease duration, where patients showed higher acceptance rates of unfair offers in the UG compared to controls (16), in the present study, patients with “deficit syndrome” did not display such a tendency. Instead, we observed generally lower acceptance rates in patients. While the overall MANOVA result was not significant, the separate ANOVA results suggested a significantly lower acceptance of fair offers in the 5:5-split condition in the UG. Further investigation indicated that both poorer ToM and higher rejection of fair offers contributed independently to discriminating patients from controls, suggesting the involvement of distinct factors. As one could suggest that this unusual behavior could be linked to distrust and paranoid ideation, we checked for correlations with PANSS items, but did not discover anything significant in this regard. Other features of deficit schizophrenia, not assessed in the current study, perhaps a more pessimistic outlook, might account for this finding.

With respect to third-party punishment of observed unfairness, earlier work found that schizophrenia patients with a mean disease duration of 5.6 years employed altruistic punishment to a similar degree compared to healthy controls, while patients with a mean disease duration of 10.8 years employed this strategy significantly less often (16, 17). Our finding that patients with deficit schizophrenia generally employed punishment significantly less than controls is consistent with these latter results. However, when controlling for ToM, we found a significant effect of ToM on third-party punishment, and a reduction of the group difference in punishment investment to a non-significant level. This suggests that this specific kind of economic decision-making, in contrast to previous work (16), is more strongly dependent on ToM in deficit schizophrenia. Unfortunately, a direct comparison of our own previous work with the present study was precluded for two reasons. First, as outlined in *Methods*, we decided to reduce the number of trials for the present study, so, a direct statistical comparison was not appropriate. More importantly, we used different ToM tasks in the two studies. That is, Wischniewski and Brüne (16) utilized the Reading the Mind in the Eyes Task (42), which some researchers consider more a test of emotion understanding than ToM (43), whereas in the present study we used a more traditional cartoon-based test of ToM reasoning (6, 31). This difference in methodology may also account for the fact that ToM had no impact on economic decision-making in Wischniewski and Brüne’s study (16), while it had more influence on third-party punishment in the current study, perhaps related to different psychometric properties of the tasks (44).

Aside from the lack of direct comparison with a group of schizophrenia patients not fulfilling the criteria for “deficit syndrome” the present study has several additional limitations. A second limiting factor of this study was the relatively small sample size and difference between groups with regard to gender. Third, potential medication effects could not be accounted for. Fourth, despite efforts to reduce the effects of the virtual nature of the economic games, we cannot rule out that task performance would have been different in ecologically more valid “real-life” conditions. Fifth, this study cannot differentiate between a potential lack of social engagement in this specific patient group, as opposed to a genuine impairment of the appreciation of rules of social exchange. Finally, it is difficult to assign the present findings solely to the chronicity or “deficit” nature of the syndrome. For example, iatrogenic effects may also have played a role, given that the participants had been hospitalized many times over the years and that the majority of them lived in residential homes for mentally ill people.

In summary, patients with chronic “deficit” schizophrenia seem to have some understanding of rules of social exchange including fairness and third-party punishment. However, in contrast to previous studies in less chronic patients, our patients with deficit schizophrenia were less likely to accept fair offers than controls, a difference that was not explained by poorer ToM in patients. Additional analysis of this unusual behavior indicated that poorer ToM and higher rejection of fair offers contributed independently to discriminating patients from controls, suggesting the involvement of distinct factors. In contrast, after adjusting for poorer ToM in our patients, the group difference in levels of investment in third-party punishment became non-significant. These findings warrant replication in larger samples and in direct comparison with less chronic states of the disorder. Future research may also need to take into consideration other cognitive factors such as executive functioning that putatively contribute to the development of a “deficit syndrome”. In a broader frame of reference, the recognition of rules of social exchange may be incorporated in programmes aiming at patient recovery and social integration.

## Data Availability Statement

The datasets generated for this study are available on request to the corresponding author.

## Ethics Statement

The studies involving human participants were reviewed and approved by Ethics Committee of the Medical Faculty of the Ruhr University Bochum, Germany. The patients/participants provided their written informed consent to participate in this study.

## Author Contributions

CS collected the data, prepared the data for statistical evaluation and wrote the first draft of the manuscript. RL contributed to the study design, refined the analyses and commented on substantial parts of the manuscript. MB designed the study, performed the statistical analyses, and re-wrote parts of the manuscript.

## Conflict of Interest

The authors declare that the research was conducted in the absence of any commercial or financial relationships that could be construed as a potential conflict of interest.
